# The importance of chest CT severity score and lung CT patterns in risk assessment in COVID-19-associated pneumonia: a comparative study

**DOI:** 10.3389/fmed.2023.1125530

**Published:** 2023-05-17

**Authors:** Miklós Szabó, Zsófia Kardos, László Kostyál, Péter Tamáska, Csaba Oláh, Eszter Csánky, Zoltán Szekanecz

**Affiliations:** ^1^Department of Pulmonology, Borsod Academic County Hospital, Miskolc, Hungary; ^2^Department of Rheumatology, Borsod Academic County Hospital, Miskolc, Hungary; ^3^Faculty of Health Sciences, University of Miskolc, Miskolc, Hungary; ^4^Department of Radiology, Borsod Academic County Hospital, Miskolc, Hungary; ^5^Department of Rheumatology, Faculty of Medicine, University of Debrecen, Debrecen, Hungary

**Keywords:** COVID-19, chest CT severity score, ground-glass opacity, survival, intensive care unit

## Abstract

**Introduction:**

Chest computed tomography (CT) is suitable to assess morphological changes in the lungs. Chest CT scoring systems (CCTS) have been developed and use in order to quantify the severity of pulmonary involvement in COVID-19. CCTS has also been correlated with clinical outcomes. Here we wished to use a validated, relatively simple CTSS to assess chest CT patterns and to correlate CTSS with clinical outcomes in COVID-19.

**Patients and methods:**

Altogether 227 COVID-19 cases underwent chest CT scanning using a 128 multi-detector CT scanner (SOMATOM Go Top, Siemens Healthineers, Germany). Specific pathological features, such as ground-glass opacity (GGO), crazy-paving pattern, consolidation, fibrosis, subpleural lines, pleural effusion, lymphadenopathy and pulmonary embolism were evaluated. CTSS developed by Pan et al. (CTSS-Pan) was applied. CTSS and specific pathologies were correlated with demographic, clinical and laboratory data, A-DROP scores, as well as outcome measures. We compared CTSS-Pan to two other CT scoring systems.

**Results:**

The mean CTSS-Pan in the 227 COVID-19 patients was 14.6 ± 6.7. The need for ICU admission (*p* < 0.001) and death (*p* < 0.001) were significantly associated with higher CTSS. With respect to chest CT patterns, crazy-paving pattern was significantly associated with ICU admission. Subpleural lines exerted significant inverse associations with ICU admission and ventilation. Lymphadenopathy was associated with all three outcome parameters. Pulmonary embolism led to ICU admission. In the ROC analysis, CTSS>18.5 significantly predicted admission to ICU (*p* = 0.026) and CTSS>19.5 was the cutoff for increased mortality (*p* < 0.001). CTSS-Pan and the two other CTSS systems exerted similar performance. With respect to clinical outcomes, CTSS-Pan might have the best performance.

**Conclusion:**

CTSS may be suitable to assess severity and prognosis of COVID-19-associated pneumonia. CTSS and specific chest CT patterns may predict the need for ventilation, as well as mortality in COVID-19. This can help the physician to guide treatment strategies in COVID-19, as well as other pulmonary infections.

## Introduction

In late 2019, severe acute respiratory syndrome coronavirus 2 (SARS CoV-2) was identified in Wuhan, China, resulting in the Coronavirus Disease 2019 (COVID-19) pandemic (1). SARS-CoV-2 virus-induced, COVID-19-associated pneumonia is a part of the 2nd–3rd stages of COVID-19 often leading to persistent airway and lung damage and consequent respiratory failure (2, 3).

Imaging measures play a crucial role in diagnostics, assessing the severity of COVID-19 pneumonia and recognizing complications. Chest computed tomography (CT) is highly sensitive in determining the presence, extent, location and nature of lung involvement, which also have prognostic significance. Among other imaging patterns, ground glass opacities (GGO), crazy-paving pattern, consolidation and honeycombing have been associated with various stages of pulmonary inflammation and fibrosis in COVID-19. Analysis and comparison of chest CT results with other clinical and laboratory parameters can help to identify high-risk patients, thereby making appropriate therapeutic decisions early (4–12).

Chest CT is also a useful tool for the assessment of the severity and prognosis of lung involvement (5, 6, 8–10, 13). Various semi-quantitative scoring tools have been proposed for CT that visually calculate the extent of lung abnormalities in COVID-19 [reviewed in (5)]. Most of these only consider the extent of lung involvement. The difference between them is mainly in the volume distribution of the lungs and the number of degrees of involvement. Pan et al. (9) proposed a semi-quantitative CT severity score (CTSS) based on the extent of lobar involvement (0–25). In the study of Francone et al. (6), CTSS was significantly higher in critical and severe compared to mild-stage patients. Moreover, CTSS positively correlated with C-reactive protein and D-dimer levels. CTSS ≥18 was associated with increased mortality risk and was predictive of death (6, 14). Deep learning and several automated softwares have been used to score chest CT scans in COVID-19 (15–17).

The sensitivity of high-resolution chest CT for detecting COVID-19 in symptomatic patients was reported to be high (67–100%), exceeding the sensitivity of RT PCR (53–88%), especially in the early stages of the disease. The specificity of CT was given in clinical studies between 25 and 80%, but it can even be in the upper third, if we consider the modest sensitivity of the RT-PCR [“reverse calculation approach”; (18)]. However, its specificity is influenced by the local prevalence of COVID-19 and other respiratory pathogenic viruses and the experience of the radiologist. Considering the radiation exposure, time requirement and potentially high number of CT scans, it is not recommended for screening but is a useful tool for supporting decisions in critically ill patients with a clinical picture of COVID but a negative RT-PCR test (18–21).

Moreover, it is known that, in addition to the degree of lung involvement, the radiological patterns and changes, which can obviously be traced back to pathological changes, have prognostic significance. A worse prognosis has been reported in the presence of consolidation, air bronchogram, pleural fluid, lymphadenomegaly and increased pulmonary artery diameter (6, 10, 15, 22).

A good solution for this could be the scoring system used in the study performed by Yuan et al. (10). This system takes into account both the extent of lung involvement and the radiological pattern. This principle is also followed by software-supported computerized scoring systems that, in addition to the lung volume, also calculate the air content of the given unit (15–17). In the study of Palumbo et al. (17), disease progression was associated with lower total lung volume and non-aerated lung tissue was related with disease progression. Although these software are very accurate and their use can save a great deal of time, they are not available in many hospitals and require radiological supervision (15–17).

We have recently performed a single-centre study in order to assess the prognosis and outcome, as well as their clinical and laboratory determinants of 233 hospital-admitted COVID-19 pneumonia patients. We also applied and found useful the A-DROP general composite scoring system (23). The A-DROP scoring system is a prognostic tool to assess the severity of community-acquired pneumonia (CAP). This 6-point scale (0–5) scores the following parameters: Age (≥70 years in males and ≥75 years in females), dehydration (BUN ≥7.5 mmoL/L), respiratory failure (SaO2 ≤ 90% or PaO2 ≤ 60 mmHg), orientation disturbance (confusion) and low blood pressure (systolic BP ≤ 90 mmHg). We used the A-DROP score in our previous published study performed in COVID-19 so we refer to this paper (23).

In the further analysis of the same cohort, we wished to determine the value of CTSS in assessing the severity and prognosis of lung involvement in our COVID-19 patients.

## Patients and methods

### Patients and study design

The major characteristics of this retrospective cohort study have been previously published (23) and also included in Table 1. Briefly, this cohort study was conducted at the Borsod Academic County Hospital, Miskolc, Hungary. SARS-CoV-2 infection was confirmed by throat-swab PCR, while pneumonia was confirmed by imaging (chest CT: 227 cases, plain X-ray: 6 cases). The criteria for hospital discharge included absence of fever for at least 3 days, cessation or significant improvement of respiratory symptoms, as well as clear improvement of the radiological picture.

**Table 1:** Patient characteristics.

Parameters at baseline	Total *N*	Mean ± SD or *N* (%)	Normal range
Age (years)	227	56.2 ± 7.8	–
Female:male ratio	227	83:144	–
Disease duration (days from first symptom)	227	8.4 ± 5.2	–
CRP (mg/l)	227	***121.8 ± 97.9***	0.2–10
Absolute WBC count (G/l)	227	8.8 ± 6.0	4.4–11.3
Absolute neutrophil count (G/l)	227	7.3 ± 7.8	2–8
Absolute lymphocyte count (G/l)	227	1.5 ± 4.3	0.8–4
Platelet count (G/l)	227	257.2 ± 108.1	150–400
PCT (ng/ml)	164	0.86 ± 7.39	0–0.5
LDH (U/l)	227	***744.2 ± 514.7***	230–460
D-dimer (ng/ml)	135	***2404.6 ± 4301.4***	0–500
ferritin (ng/ml)	122	***1202.7 ± 1921.7***	20–300
IL-6 (pg/ml)	66	***129.7 ± 138.0***	0–7
BUN (mmol/l)	227	6.5 ± 4.5	2.9–8.5
creatinine (μmol/l)	227	97.2 ± 89.5	64–104
Fever	227	144 (63.4)	–
Dyspnoea	227	157 (69.2)	–
Coughs	227	161 (70.9)	–
Confusion/dizziness	227	9 (4.0)	–
PaO_2_ (mmHg)	199	58.4 ± 16.2	80–100^*^
SaO_2_ (%)	227	89.5 ± 7.7	95–99^*^
systolic BP (mmHg)	227	139.4 ± 23.3	90–140^*^
A-DROP	227	0.93 ± 0.78	0–1^*^
Immunosuppressive therapy (current)	227	16 (7.1)	–
Smoking (current)	66	12 (18.2)	–
CTSS	**227**	**14.6 ± 6.7**	**0**
**Medical history**	**Total *N***	***N* (%)**	
Hypertension (history)	227	149 (65.6)	–
CAD (history)	227	50 (22.0)	–
Stroke (history)	227	14 (6.2)	–
CKD (history)	227	12 (5.3)	–
Diabetes mellitus (history)	227	62 (27.3)	–
Obesity (history)	227	69 (30.4)	–
Malignancy (history)	227	10 (4.4)	–
COPD/asthma (history)	227	49 (21.6)	–
**Outcome measures**	**Total *N***	**Mean ± SD or *N* (%)**	
Time of hospitalization (days)	227	12.2 ± 6.9	–
ICU admission	227	48 (21.1)	–
Need for ventilation	227	45 (19.8)	–
Need for NIV	227	9 (4.0)	–
Need for IV	227	36 (15.6)	–
Deaths	227	39 (17.2)	–

* Age-dependent. Significantly elevated mean values are in bold italics. A-DROP, Age, Dehydration, Respiratory failure, Orientation disturbance (confusion) and low blood Pressure; BP, blood Pressure; BUN, blood urea nitrogen; CAD, coronary artery disease; CTSS, chest CT Severity Score; CKD, chronic kidney disease; COPD, chronic obstructive pulmonary disease; CRP, C-reactive protein; ICU, intensive care unit; IL, interleukin; IV, invasive ventilation; LDH, lactate dehydrogenase; NIV, non-invasive ventilation; PaO_2_, partial oxygen pressure; PCT, procalcitonin; SaO_2_, oxygen saturation; WBC, white blood cell.

The Ethics Committee of the Borsod Academic County Hospital approved this study (BORS 04/2021). We conducted this study according to the Declaration of Helsinki.

### Clinical, laboratory and imaging data collection

We reviewed all clinical electronic medical records, laboratory reports, as well as chest CT and X-ray images and collected a set of data as described previously (23). The CTSS and specific pathologies were correlated with demographic, clinical and laboratory data, as well as A-DROP scores (11). Chest CT assessment was performed by three qualified radiologists (LK, PT, OCS). Radiologists were blinded from the clinical data. All data were evaluated by two physicians (MS, ZK) and a third researcher (ZS) adjudicated any difference in interpretation between the two primary reviewers.

### Chest CT scan protocol

Chest CT scans were performed using a single inspiratory phase in a 128 multi-detector CT scanner (SOMATOM Go Top, Siemens Healthineers, Germany). To minimize motion artifacts, patients were instructed on breath-holding; CT images were then acquired during a single breath-hold. For CT acquisition, the tube voltage was 90 kVp with automatic tube current modulation. From the raw data, 1 mm slices were reconstructed with a pulmonary Br 64 kernel and a mediastinal Br40 kernel (Siemens Healthineers, Germany) in all three planes. All thin-section CT images were reviewed at a window width and level of 400 and 40 HU and 1,200 and −600 HU respectively, for the mediastinum and lung parenchyma.

### CT image analysis to quantify the extent of pulmonary involvement

On the chest CT, the specific pathological features, such as ground-glass opacity (GGO), crazy-paving pattern, consolidation, fibrosis, subpleural lines, pleural effusion, lymphadenopathy and pulmonary embolism were also evaluated, based on the Fleischner Society Nomenclature recommendations (24) and previous COVID-19-related radiology publications (25, 26).

For quantitative scoring (CTSS) we used the protocol first published by Pan et al. (9) and adapted it to our patients. We will refer to this protocol as “CTSS-Pan.” In this analysis, we used the chest CT images of 227 patients and calculated the Chest CT severity score based on 3 methods. CTSS was calculated for all 227 patients according to Pan et al. (9). In brief, the extent of anatomic involvement was calculated in each of the 5 lobes. In each lobe, the absence of lobar involvement (0%) yielded to a score of 0, while <5%, 5–25%, 26–50%, 51–75 and >75% involvement was scored as 1, 2, 3, 4 and 5, respectively. Thus, the individual scores of the five lobes resulted in a global score of 0 to 25.

In the case of 98 patients, two other validated scoring systems published by Yang et al. (14) and by Yuan et al. (10) were also used. We compared our results applying the CTSS-Pan protocol with these two other systems further referred to as “CTSS-Yang” and “CTSS-Yuan,” respectively. In the CTSS-Yang protocol reported by Yang et al. (14), CT features, such as GGO, interstitial opacity and air trapping were determined and correlated with clinical and laboratory parameters. The 18 anatomic segments of both lungs were divided into 20 regions and lung pathologies were associated with scores of 0, 1 or 2 in each region. Thus CTSS-Yang may range from 0 to 40 points (14). In the CTSS-Yuan protocol published by Yuan et al. (10), the extent of involvement of each abnormality was assessed independently for each of 3 zones: upper (above the carina), middle (below the carina and above the inferior pulmonary vein), and lower (below the inferior pulmonary vein). The CT findings were graded on a 3-point scale (1: normal; 2: ground-glass attenuation; 3: consolidation). Each lung zone, with a total of six lung zones in each patient, was assigned a scale according to distribution of the affected lung parenchyma (0, normal; 1: <25%; 2: 25–50%; 3: 50–75%; 4: >75% abnormality). The four-point scale of the lung parenchyma distribution was then multiplied by the radiologic scale described above. Points from all zones were added for a final total cumulative score, with value ranging from 0 to 72 (10).

We compared CTSS-Pan applied to 227 to patients to the other two scoring systems (CTSS-Yang and CTSS-Yuan).

### Statistical analysis

Statistical analysis was performed using the SPSS software v.28.0 (IBM, Armonk, NY, United States). Data are expressed as mean ± SD for continuous and case number plus percentages (*n*, %) for categorical variables. The distribution of continuous variables was determined by Kolmogorov–Smirnov test. Continuous variables (e.g., CTSS scores) were assessed by Mann–Whitney U-tests. Nominal variables were compared by *χ*^2^ or Fisher’s exact test. Spearman’s analysis was used to test for correlations. Multiple comparisons were performed using the stepwise method. Multivariable regression analysis was performed in order to assess determinants of outcome parameters as dependent variables. Receiver Operating Characteristic (ROC) curves show the sensitivity and specificity for every possible cut-off for a test. The cut-off value was set where the sum of sensitivity and specificity was the highest. Area under the ROC curve is measure of the usefulness of a characteristic, where a greater area means a more useful test. Odds ratio (OR), negative (NPV) and positive predictive values (PPV) were calculated with respect to clinical outcomes. *p* < 0.05 were considered significant in all tests mentioned above.

## Results

### Characterization of patients

The major characteristics of the full patient cohort (*n* = 233) have been published (23). The characteristics of the 227 patients that underwent chest CT are included in Table 1. The 227 patients included 144 men and 83 women. Their mean age was 56.2 ± 7.8 years (range: 40–76 years). Disease duration was 8.4 ± 5.2 days (range: 1–35 days). Altogether 18.2% were current smokers and 7.1% received immunosuppressive drugs. Among the patients 65.6% had hypertension, 22.0% had CAD, 6.2% had stroke, 5.3% had CKD, 27.3% had diabetes mellitus, 30.4% had obesity, 4.4% had malignancies and 21.6% had COPD/asthma in their history. At the time of admission, about 63–71% of patients had fever, dyspnoea and/or coughs, while 4.0% had confusion/dizziness (Table 1). With respect to lab results, at admission most of these patients had elevated CRP, ferritin, D-dimer, LDH and IL-6 levels (Table 1). Out of the 227 included patients, 48 (21.1%) had to be admitted to ICU. Forty-five patients (19.8%) needed ventilation. Out of them, 9 (4.0%) required non-invasive (NIV) and 36 (15.6%) invasive ventilation (IV). Altogether 39 patients (17.2%) died. The duration of hospitalization was 12.2 ± 6.9 days (range: 2–48 days; Table 1). The mean A-DROP score within these 227 patients was 0.93 ± 0.78 (Table 1).

### CTSS and chest CT patterns may be a useful tool to determine the severity and prognosis of pneumonia

The mean CTSS in the 227 COVID-19 patients was 14.6 ± 6.7 (Table 1). In the binary analysis, the need for ICU admission (*p* < 0.001) and death (*p* < 0.001) were significantly associated with higher CTSS (Table 2). Ventilation also more commonly had to be administered to patients with higher CTSS (*p* < 0.001; Table 2). With respect to NIV and IV, both the need for NIV (*p* = 0.014) and that for IV (*p* < 0.001) versus no need for ventilation were significantly associated with higher CTSS (data not shown). In contrast, CTSS could not differentiate between patients in need for NIV versus IV (data not shown).

**Table 2:** The association of CCTS-Pan and chest CT patterns with ICU admission, need for ventilation and death.

Parameter	Number of patients out of the total of 227 with the given parameter (%)	ICU admission (Y/N)	Ventilation (Y/N)	Death (Y/N)
		*p* value		
CCTS-Pan	227 (100)	**<0.001**	**<0.001**	**<0.001**
GGO	155 (68.3)	0.679	0.698	0.794
Crazy paving pattern	87 (38.3)	**0.023**	0.062	0.851
Consolidation	78 (34.4)	0.579	0.141	**0.031**
Fibrosis	4 (1.8)	0.373	0.711	0.427
subpleural lines	33 (14.5)	**0.006**	**0.039**	0.089
pleural effusion	19 (8.4)	0.968	0.884	0.712
lymphadenopathy	88 (38.8)	**0.041**	**0.044**	**0.049**
pulmonary embolism	13 (5.7)	**0.021**	0.135	0.114

Mann–Whitney U test. Significant differences are in bold italics. CCTS, chest CT Severity Score; GGO, ground glass opacity; ICU, intensive care unit; N, no; Y, yes.

With respect to chest CT patterns, GGO, crazy-paving pattern, consolidation, fibrosis, subpleural lines, pleural effusion, lymphadenopathy and pulmonary embolism were observed in 68.3, 38.3, 34.4, 1.8, 14.5, 8.4, 38.8 and 5.7% of the patients, respectively (Table 2). Crazy-paving pattern was significantly associated with ICU admission, but not with the need for ventilation or death. Subpleural lines exerted significant associations with ICU admission and ventilation. Consolidation correlated with death. Lymphadenopathy was associated with all three outcome parameters. Pulmonary embolism led to ICU admission. GGO, fibrosis and pleural effusion did not show any associations with any outcome measures, however, the number of patients with fibrosis and pleural effusion was small (Table 2).

**Figure 1:**
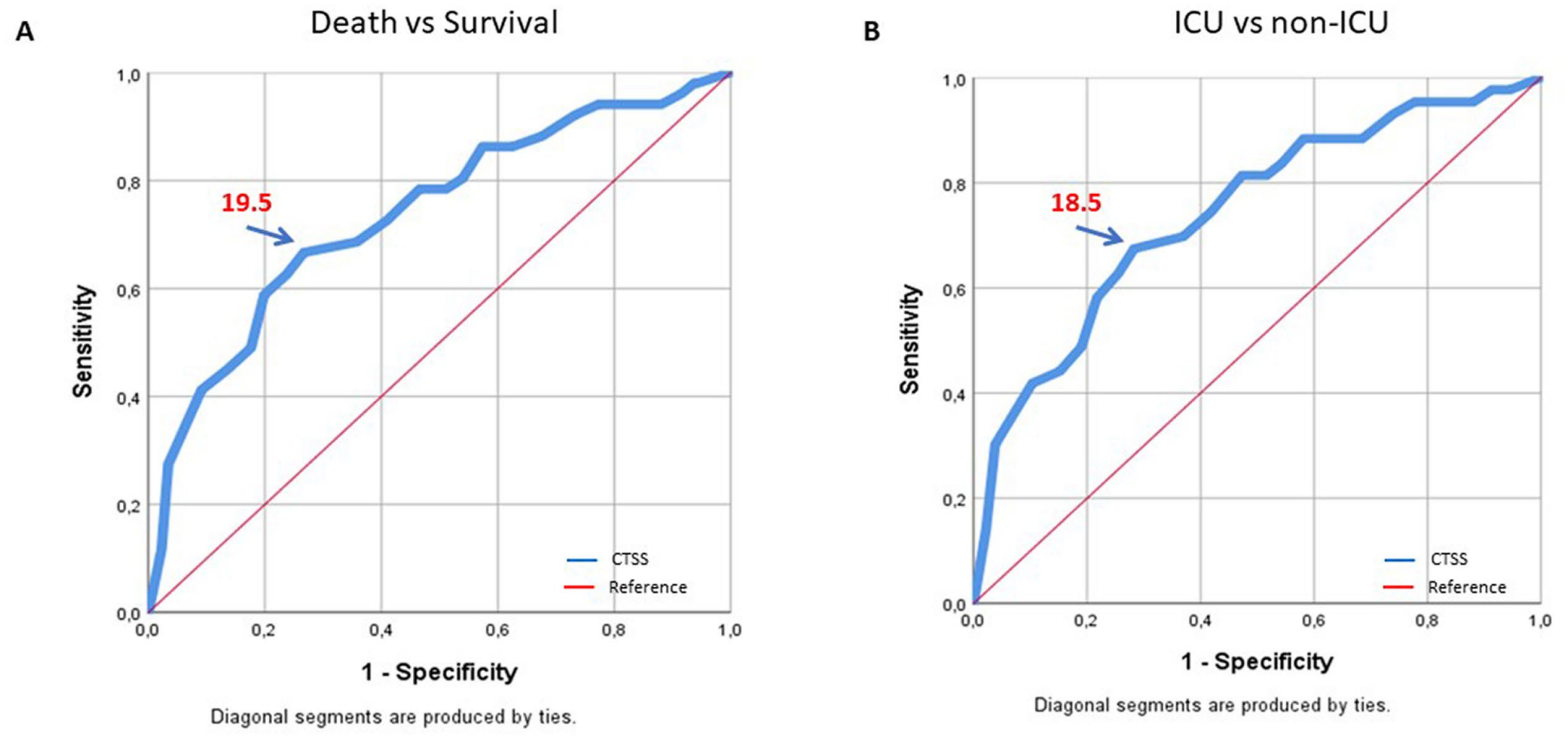
ROC curve analysis of the association of CTSS-Pan values with death versus survival **(A)** and the need for ICU admission **(B)** in COVID-19 patients.

In the ROC analysis, CTSS>18.5 significantly predicted admission to ICU (*p* = 0.026) and CTSS>19.5 was the cutoff for increased mortality (*p* < 0.001; Table 3; Figure 1). This cutoff has a sensitivity and specificity of about 60–70%.

**Table 3:** ROC curve data of the association of CCTS-Pan with ICU admission and death.

Parameter	ICU admission (Y/N)					Death (Y/N)				
	Cutoff	Sens.	Spec.	ROC Area	*p* value	Cutoff	Sens.	Spec.	ROC Area	*p* value
CCTS	18.5	0.628	0.739	0.609 ± 0.050	**0.026**	19.5	0.618	0.767	0.728 ± 0.049	**<0.001**

ROC analysis was performed. Significant differences are in bold italics. CCTS, chest CT Severity Score; N, no; Sens., sensitivity; Spec., specificity; Y, yes.

### Correlations of CTSS with other parameters

In the simple Spearman’s correlation analysis, CTSS significantly and positively correlated with age, absolute leukocyte and platelet counts, CRP, PCT, LDH, D-dimer, ferritin, IL-6, BUN and the A-DROP score. CTSS showed inverse correlation with total lymphocyte count, PaO2 and SaO2 (Table 4).

**Table 4:** Significant correlations of CCTS-Pan with other parameters.

Parameter	CCTS	
	*R* value	*p* value
Age	0.187	**0.005**
Absolute WBC count	0.235	**<0.001**
Absolute lymphocyte count	−0.150	**0.024**
Absolute platelet count	0.138	**0.007**
CRP	0.545	**<0.001**
PCT	0.397	**<0.001**
LDH	0.549	**<0.001**
D-dimer	0.230	**0.008**
Ferritin	0.312	**0.001**
IL-6	0.294	**0.020**
BUN	0.197	**0.003**
PaO2	−0.388	**<0.001**
SaO2	−0.467	**<0.001**
A-DROP	0.322	**0.002**

Spearman’s correlation analysis. Significant correlations are in bold italics. A-DROP, Age, Dehydration, Respiratory failure, Orientation disturbance (confusion) and low blood Pressure; BUN, blood urea nitrogen; CCTS, chest CT Severity Score; CRP, C-reactive protein; IL, interleukin; LDH, lactate dehydrogenase; PaO2, partial oxygen pressure; PCT, procalcitonin; SaO2, oxygen saturation; WBC, white blood cell.

A multivariable regression analysis was performed in order to determine the correlations of outcome parameters with others (Table 5). The need for admission to ICU was associated with the A-DROP score and obesity (*p* < 0.05). The need for ventilation was determined by CCTS-Pan, A-DROP and obesity (*p* < 0.05). Finally, the risk factors for death were obesity, neutrophil counts, PCT and BUN (*p* < 0.05; Table 5).

**Table 5:** Multivariable regression analysis^*^ of outcome indicators.

Dependent variable	Independent variable	OR	95% CI	*p* value
Admission to ICU	A-DROP score	4.885	1.574–14.930	**0.006**
	Obesity	3.839	1.419–10.385	**0.008**
Need for ventilation	CCTS-Pan	3.348	1.096–10.229	**0.034**
	A-DROP score	8.519	2.520–28.804	**0.001**
	Obesity	3.643	1.238–10.715	**0.019**
Death	Obesity	1.666	0.632–6.948	**0.008**
	Neutrophil count	0.044	0.021–4.225	**0.040**
	PCT	1.872	0.822–5.184	**0.023**
	BUN	0.165	0.082–4.075	**0.044**

* Binary logistic regression, Forward LR method. Significant correlations are in bold italics. A-DROP, Age, Dehydration, Respiratory failure, Orientation disturbance (confusion) and low blood Pressure; BUN, blood urea nitrogen; CCTS, chest CT Severity Score; CI, confidence interval; ICU, intensive care unit; OR, Odds ratio; PCT, procalcitonin.

### Comparison of three scoring systems

After performing the study by using the CTSS-Pan system (9), we compared the performance of this system to both CTSS-Yang (14) and CTSS-Yuan (10). When performing Spearman’s correlation analysis between any two systems, CTSS-Pan correlated with CTSS-Yang (*R* = 0.899, *p* < 0.001), CTSS-Pan correlated with CTSS-Yuan (*R* = 0.909 *p* < 0.001) and CTSS-Yang correlated with CTSS-Yuan (*R* = 0.928, *p* < 0.001; data not shown).

**Figure 2:**
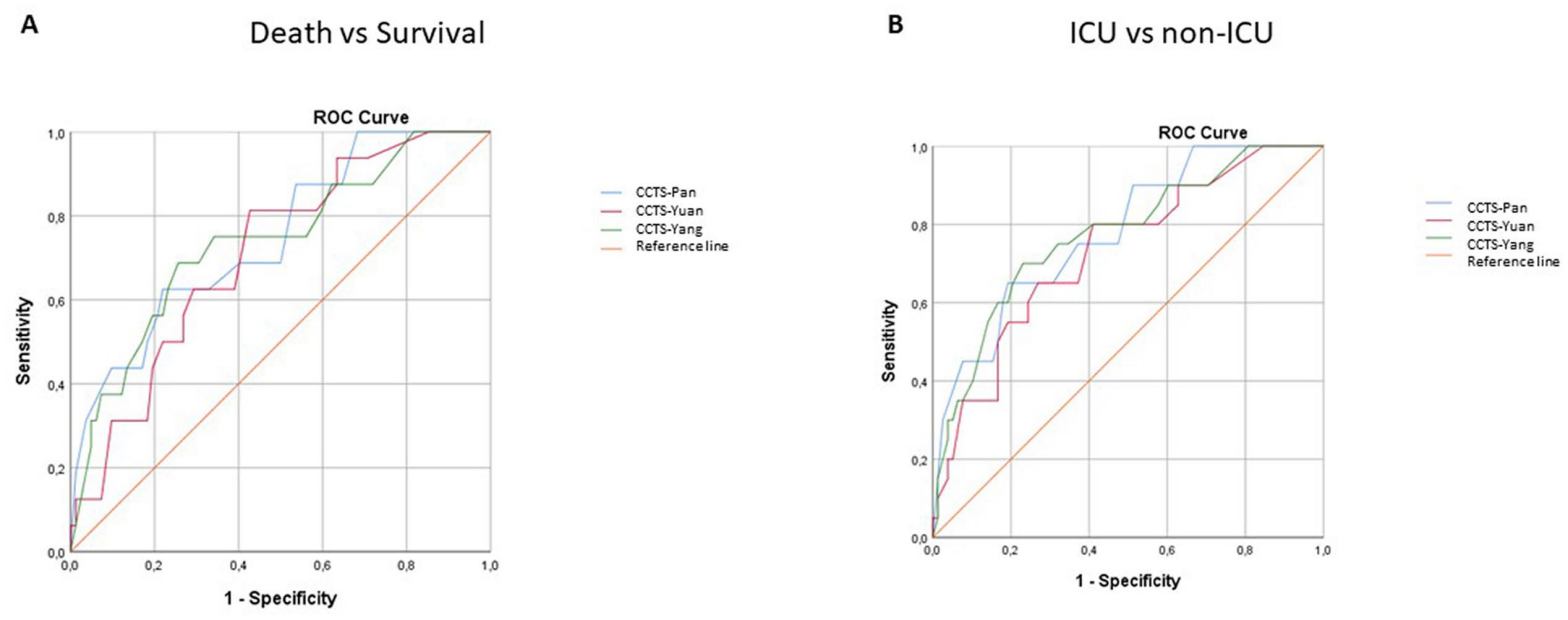
ROC curve analysis of the association of three CTSS systems with death versus survival **(A)** and the need for ICU admission **(B)** in COVID-19 patients.

With respect to outcome, survival versus death and ICU admission versus non-ICU were also analyzed (Table 5). According to the Mann–Whitney test, all three CTSS systems could significantly differentiate between patients who survived or died and between those who required ICU admission and those who did not (Table 6A). Figure 2 shows the ROC analysis of the comparison of the 3 systems with respect to survival versus death (Figure 2A) and ICU versus non-ICU (Figure 2B). There were no differences between the performances of CTSS-Pan, CTSS-Yang and CTSS-Yuan. As shown in Table 5, by using *χ*^2^ or Fisher’s exact tests, we could determine cut-off values that were able to differentiate between favorable and non-favorable outcomes. With respect to CCTS-Pan, CCTS-Yang and CCTS-Yuan, these cutoff values were 17, 23 and 28, respectively (Table 6B). The OR (95% CI) values of death versus survival were 5.9, 6.0 and 3.1, respectively, while these for ICU versus non-ICU were 7.8, 7.2 and 4.1, respectively (Table 6B). Among the 3 systems, CTSS-Pan had the highest PPV with respect to both death versus survival and ICU versus non-ICU. CTSS-Pan also performed well regarding NPV. Among the 82 patients who survived, CCTS-Pan (*R* = 0.407, *p* < 0.001), CCTS-Yang (*R* = 0.353, *p* = 0.001) and CCTS-Yuan (*R* = 0.385, *p* < 0.001) exerted low-grade but significant correlations with length of hospital stay in days (data not shown).

**Table 6:** Comparison of three CTSS systems with respect to survival versus death and ICU admission versus non-ICU.

A. Performance of the 3 CTSS systems in differentiating between survival versus death and non-ICU versus ICU						
Scoring system	Survived (*n* = 82)	Died (*n* = 16)	*p* value	non-ICU (*n* = 78)	ICU (*n* = 20)	*p* value
CCTS-Pan	12.6 ± 6.4	18.4 ± 6.1	**0.002**	12.2 ± 6.3	18.0 ± 5.7	**<0.001**
CCTS-Yang	19.3 ± 8.7	27.7 ± 9.4	**0.003**	18.7 ± 8.4	28.2 ± 9.0	**<0.001**
CCTS-Yuan	23.1 ± 13.2	33.1 ± 14.5	**0.008**	22.3 ± 12.7	33.9 ± 14.7	**0.002**

B. Determination of cut-off values with respect to predicting outcomes												
Scoring system	Survived (*n*)	Died (*n*)	*p* value	OR (95%CI)	PPV	NPV	non-ICU (*n*)	ICU (*n*)	*p* value	OR (95%CI)	PPV	NPV
CCTS-Pan >17	18	10	**0.002**	5.9 (1.9–18.5)	0.357	0.914	15	13	**<0.001**	7.8 (2.7–22.9)	0.464	0.900
CCTS-Pan ≤17	64	6					63	7				
CCTS-Yang >23	22	11	**0.001**	6.0 (1.9–19.2)	0.333	0.923	19	14	**<0.001**	7.2 (2.4–21.5)	0.424	0.908
CCTS-Yang ≤23	60	5					59	6				
CCTS-Yuan >28	24	9	**0.037**	3.1 (1.0–9.3)	0.273	0.892	21	12	**0.005**	4.1 (1.5–11.3)	0.364	0.877
CCTS-Yuan ≤28	58	7					57	8				

Significant differences are in bold italics. CCTS, chest CT Severity Score; CI, confidence interval; ICU, intensive care unit; OR, odds ratio; NPV, negative predictive value; PPV, positive predictive value.

## Discussion

Chest CT and its scoring is very useful in determining pulmonary involvement of COVID-19 (4–11). In our single-centre study, 233 COVID-19 patients were admitted to hospital (23) and 227 patients had chest CT scans. Here we studied chest CT patterns and pathologies and calculated the CTSS semi-quantitative score. We correlated these CT findings with outcome measures, such as need for ICU admission, ventilation and death, as well as various clinical, laboratory parameters and the A-DROP composite score (23). We found CTSS a useful composite score in order to predict the need for ventilation and death in COVID-19-associated pneumonia. Indeed, among several other factors, CTSS-Pan showed the highest significance when determining outcome parameters. Moreover, some CT patterns and pathologies may also be associated with clinical outcomes.

The mean CTSS of our patients was about 14.6, which can be considered as moderate–severe pneumonia. In the study of Francone et al. (6), CTSS ≥18 was associated with higher mortality. Yang et al. (14) also found higher CTSS in sever compared to mild COVID-19. Furthermore, the optimal threshold to identify severe COVID-19 pneumonia was 19.5 (14). In our study, CTSS was associated with the need for admission to ICU and ventilation, as well as death. Moreover, according to the ROC analysis, the cutoff values for ICU admission and death were 18.5 and 19.5, respectively. This is similar to other studies (6, 14).

CTSS significantly and positively correlated with age, absolute leukocyte and platelet counts, CRP, PCT, LDH, D-dimer, ferritin, IL-6, BUN and the A-DROP score, while negatively correlated with total lymphocyte count, PaO2 and SaO2. This is in accordance with the value of these biomarkers in COVID-19. Advanced COVID-19 is associated with systemic inflammation and cytokine storm. Inflammatory biomarkers, such as CRP, PCT, D-dimer, ferritin and IL-6 are abundantly produced in stage 2b-3 of COVID-19 (2, 3, 27–29). Severe COVID-19 is also associated with leukocytosis and lymphopenia (27–29). Elevated LDH is a marker of lung injury (3, 28, 30). Increasing lung injury is certainly correlated with decreasing PaO2 and SaO2 (9, 30). Francone et al. (6) also reported correlations of CTSS with CRP and D-dimer.

According to various chest CT patterns, we found significant positive associations between crazy-paving pattern and lymphadenopathy with the need for ICU admission and ventilation, as well as with death in COVID-19. In the study of Francone et al. (6) GGO was predominant in the early-phase (≤7 days since symptoms’ onset), while crazy-paving pattern, consolidation, and fibrosis characterized late-phase disease (>7 days). These authors did not correlate CT patterns with ICU admission, ventilation and death. However, Martinez Chamorro et al. (5) consider crazy-paving pattern and consolidation as markers of disease progression. On the other hand, as GGO is predominant in the early, moderate stage of COVID-19 (5, 6, 10), our result showing that GGO did not correlate with any of the three outcome measures seems to be underscored. In contrast, the presence of subpleural lines showed inverse association with the need for ICU admission and worse outcome. Thus, subpleural lines might indicate better prognosis. The explanation could be that those having subpleural lines are already past the critical stage and would probably survive. Thus, crazy-paving pattern, consolidation, subpleural lines and lymphadenopathy may be clinically useful markers for clinical outcome including ICU admission, ventilation and death. Other investigators also found CTSS useful in determining the clinical outcome of COVID-19 (31, 32).

When denominators of outcome parameters were determined, mostly CTSS-Pan, and, to a lesser extent, the A-DROP-score and obesity determined the need for ICU admission, ventilation and death.

We also compared the CTSS-Pan system used by us with two other systems, CTSS-Yang and CTSS-Yuan. According to paired correlations and ROC curve analysis, the performance of the three CTSS systems were highly similar. Moreover, all three systems could be used to predict death versus survival, ICU admission versus non-ICU, as well as duration of hospitalization. We could also determine optimal cutoff values for these differentiations. Yet, according to the calculation of OR and PPV, the performance of CTSS-Pan in determining outcomes was slightly better than that of CTSS-Yang or CTSS-Yuan. There have been very few studies that compared multiple CTSS systems in COVID-19. Elmokadem et al. (33) compared the diagnostic performance of 5 different CT chest severity scoring systems for COVID-19 including chest CT severity score (CTSS), chest CT score (CTS), total severity score (TSS), modified total severity score (m-TSS) and 3-level chest CT severity score (3 L-CTSS). These authors also concluded that all CTSS systems demonstrated reasonable performance to assess COVID-19 in relation to the clinical severity. CTSS and TSS had the highest specificity and least time for interpretation.

This study has certain strengths and limitations. The major strength of this study is that this is one of the first relatively large studies assessing the value of CTSS and chest CT patterns in a complex way, in association with a number of clinical, laboratory and outcome markers. The possible limitations may include the single-center nature of the study, the relatively small number of subjects and the retrospective nature of the study. In addition, except for comparing the three scoring systems, we have not validated our results against other studies and we have not addressed possible population-specific biases.

In conclusion, CTSS may be suitable to assess severity and prognosis of COVID-19-associated pneumonia. CTSS and specific chest CT patterns may predict the need for ICU admission and ventilation, as well as mortality in COVID-19. This can help the physician to guide treatment strategies in pulmonary manifestation of infectious-inflammatory diseases, such as COVID-19.

## Data availability statement

The raw data supporting the conclusions of this article will be made available by the authors, without undue reservation.

## Ethics statement

The studies involving human participants were reviewed and approved by the Ethics Committee of the Borsod Academic County Hospital approved this study (BORS 04/2021). We conducted this study according to the Declaration of Helsinki. The patients/participants provided their written informed consent to participate in this study.

## Author contributions

MS: conceptualization, investigation, patient recruitment, data analysis, and manuscript draft and finalization. ZS, PT, and CO: investigation and patient recruitment. LK: imaging, investigation, and imaging data analysis. EC: supervision and patient recruitment. ZS: supervision, conceptualization, data analysis, manuscript draft and finalization, and financial support. All authors contributed to the article and approved the submitted version.

## Conflict of interest

The authors declare that the research was conducted in the absence of any commercial or financial relationships that could be construed as a potential conflict of interest.

## Publisher’s note

All claims expressed in this article are solely those of the authors and do not necessarily represent those of their affiliated organizations, or those of the publisher, the editors and the reviewers. Any product that may be evaluated in this article, or claim that may be made by its manufacturer, is not guaranteed or endorsed by the publisher.
